# Supporting Adolescents: Perceived Parental Support Associated with Decreased Adolescent Loneliness and Emotion Suppression in a National Sample

**DOI:** 10.3390/children12091108

**Published:** 2025-08-23

**Authors:** Alec Qualitza, Chan L. Thai, Jasmín D. Llamas

**Affiliations:** 1 Departments of Psychology and Public Health, Santa Clara University, 500 El Camino Real, Santa Clara, CA 950503, USA; aqualitza@alumni.scu.edu; 2 Department of Communication, Santa Clara University, 500 El Camino Real, Santa Clara, CA 950503, USA; cthai@scu.edu; 3 Department Counseling Psychology, Santa Clara University, 500 El Camino Real, Santa Clara, CA 950503, USA

**Keywords:** parental responsiveness, emotional well-being, psychosocial factors, loneliness, emotion regulation, adolescent development

## Abstract

**Highlights:**

What are the main findings?

- Responsive parenting is associated with decreased emotion suppression in adolescents.
- Responsive parenting is associated with decreased loneliness among adolescents.

What is the implication of the main finding?

- Interventions to promote responsive parenting may lead to improved adolescent health.

**Abstract:**

**Background:** Parenting styles are associated with various health outcomes among children, such as fruit and vegetable consumption, physical activity, and emotional well-being. Previous literature suggests that responsive parenting styles are associated with improved mental health outcomes. This study examines the association between an adult’s responsive parenting style and the psychosocial outcomes of emotion regulation and loneliness among children. **Methods:** Using data from the U.S. National Cancer Institute’s Family Life, Activity, Sun, Health, and Eating (FLASHE) cross-sectional survey, this secondary data analysis study seeks to understand how perceived parental responsiveness is associated with adolescents’ emotional health outcomes from the perspective of the adolescent. The sample consisted of 12- to 17-year-olds (n = ~1560), mostly female (50.2%) and non-Hispanic White (63.7%), with a mean age of 14.48 years (SD = 1.61). **Results:** Through multivariable regression, we found that perceived parental responsiveness negatively predicted adolescent emotion suppression (β = −0.29, *p* < 0.001), even after controlling for sex, age, race/ethnicity, parental marital status, parental education level, parent’s time spent at work, and the number of kids in the household (β = −0.29, *p* < 0.001). We also found that perceived parental responsiveness was the strongest predictor for adolescent loneliness (β = −0.27, *p* < 0.001), even when controlling for sex, age, race/ethnicity, parental marital status, parental education level, parent’s time spent at work, and the number of kids in the household (β = −0.28, *p* < 0.001). **Conclusions:** These results suggest that implementing interventions targeting parental responsiveness may be beneficial to improving adolescents’ emotional well-being.

## 1. Introduction

Adolescence is a formative time of change and development that presents a critical period of vulnerability. Almost half of all teens in the U.S. have at least one mental health disorder [1]. While the role of parents begins to shift during this period, their presence as a predictive and protective factor for a variety of outcomes remains (e.g., delinquency, well-being, depression, physical activity, and screen time) [2,3]; thus, strong relationships with parents may serve an important role in adolescent well-being. Many factors influence adolescent psychopathology, and among these determinants, psychosocial factors relating to emotion management and perceived social isolation are notable [4,5]. Parents’ supportive role in fostering emotional well-being in adolescence can improve health outcomes later in life and help to prevent the development of maladaptive social and emotional behaviors [6]. This study explores the role of adolescent perceived parental responsiveness in emotional suppression and loneliness.

The literature on the positive impact of parental support, a facet of parental responsiveness, on adolescent psychological outcomes and adjustment is extensive and robust [7,8]. Parental responsiveness refers to the affection, involvement, and support parents provide to their children and is an important socialization process within a family. This can be especially important for adolescents as they engage in the paradoxical tasks of seeking independence from parents while also striving to remain connected. Parental responsiveness has been identified as an important predictor for risky behaviors such as drinking, smoking, and drug use [9,10,11]. Conversely, parental conflict related to rejection or criticism is linked to poorer adolescent psychological adjustment [12,13]. The prevailing research demonstrates that despite teens’ emerging independence from parents, parental support continues to be critical in healthy psychological adjustment [14].

Other literature suggests that parental responsiveness may impact the way children regulate emotions, particularly around negative emotions [15]. Emotion regulation captures the ways in which we seek to influence our own and others’ emotions [16,17]. Emotional suppression is the active inhibition of emotional expression [17,18,19]. Emotion suppression can be socially beneficial in providing self-control of emotion-expressive behaviors in certain social contexts [20,21,22]. However, this is also a cognitively demanding process that decreases our positive emotional experiences while also not significantly affecting the way we experience negative emotions [23]. Emotion suppression may be linked to decreased social support from others, as those who suppress are less likely to share both their positive and negative emotions with others, thereby curbing the potential for support [23,24,25].

As teens begin to distance themselves psychologically from their parents, they are concurrently searching for their place in the social world, which can feel lonely [26]. Loneliness, or the experience of feeling left out or abandoned, is especially prevalent during adolescence. Loneliness goes beyond physical isolation, as lonely and non-lonely individuals do not show differences in time spent interacting with others; however, for individuals who report loneliness, these social interactions are lower in quality and provide individuals with less comfort and support [27]. Adolescence is a sensitive period where the risk for loneliness is not only increased but may also have developmental consequences that impact social skills and attitudes well into adulthood [28]. Loneliness can impact one’s cognition, affect, attention, and behaviors and can lead to negative physical and mental health outcomes [29]. Loneliness has been correlated with psychosocial outcomes such as lower self-esteem and a decreased sense of social well-being, as well as mental health issues including anxiety, depression, and suicidality [28]. Globally, adolescent loneliness has been on the rise and is a risk factor for overall health and well-being spanning beyond adolescence into adulthood [30,31,32,33]. This has been especially true during the COVID-19 pandemic, which caused reductions in physical and social contact and increased isolation and loneliness resulting in psychological distress [34,35]. Existing literature has found parental support to help mitigate the harmful ramifications of loneliness as it has been found to help offset the risk of poor self-rated health and diagnosed depression [5]. Social support in multiple contexts in adolescence is linked with improved socioemotional well-being; however, parental support during this time can serve as a protective factor against loneliness more so than other domains of social support as parents may be better equipped to discern symptoms of loneliness in their children [21].

The aim of this study is to investigate the role perceived parental responsiveness plays in predicting how adolescents regulate their emotions and their self-reported levels of loneliness using a national dataset. We hypothesize that increased parental responsiveness from at least one parent will be associated with decreased emotional suppression and decreased loneliness. By utilizing a national dataset, the findings from this study may inform the design of parenting interventions to improve adolescent mental health with greater generalizability.

## 2. Materials and Methods

The present secondary data analysis study used data obtained from the US National Cancer Institute’s Family Life, Activity, Sun, Health and Eating (FLASHE) Survey. The FLASHE study was an internet-based, cross-sectional survey conducted through Westat Inc. from April to October 2014. It assessed psychosocial, generational, and environmental correlations of cancer-preventive behaviors for parent–adolescent dyads. The FLASHE study had a 38.7% response rate with a total of 1945 dyads enrolled in the study and was approved by NCI’s and Westat’s Institutional Review Boards in 2013. Further study methodology and more extensive survey development information are provided elsewhere [36]. All adolescents who started a survey completed it in its entirety and there were no partially completed surveys, resulting in a total of 1590 adolescents that completed both FLASHE surveys. Though FLASHE data can be used to conduct dyadic-level analyses, the present study conducts individual-level analyses to investigate how adolescents’ perception of their parents’ responsiveness impacts specific psychosocial outcomes.

### 2.1. Measures

#### 2.1.1. Perceived Parental Responsiveness

Adolescents responded to six items from a modified version of the 15-item Parenting Style Inventory II (PSI-II) [37]. The PSI-II assesses three parenting style dimensions: emotional responsiveness, psychological autonomy-granting, and demandingness. Two items were used in the present study to measure emotional responsiveness items as an indicator of the adolescent’s perceived support from their parents [3,38]. Adolescents were asked to think about their relationship with their parent(s) and rate their level of agreement with the following statements: “I can count on my parent(s) to help me out if I have a problem” and “My parent(s) don’t like me to tell them my troubles” (1 = Strongly disagree, 5 = Strongly agree). The latter item was reverse-scored. An aggregate variable was created from the mean score of the summed items (α = 0.55) with a similar moderate correlation, as found in a previous study (α = 0.41, *p* < 0.0001) [3].

#### 2.1.2. Emotional Suppression

To assess the extent to which they regulated their emotions, adolescents answered four items drawn from the Suppression factor of the Emotion Regulation Questionnaire [39]. They reported their level of agreement with the following statements: “I keep my emotions to myself”; “When I am feeling POSITIVE emotions, I am careful not to express them”; “I control my emotions by NOT EXPRESSING THEM”; and lastly, “When I am feeling NEGATIVE emotions, I make sure not to express them” (1 = Strongly disagree, 5 = Strongly agree). Higher scores on these items suggest adolescents suppress their emotions more. The four items were summed, and a mean score was calculated to create an aggregate variable with an acceptable reliability score (α = 0.80).

#### 2.1.3. Loneliness

To evaluate perceived loneliness and social isolation, the FLASHE survey included two items from the UCLA Loneliness Scale [40]. Adolescents reported how often the statements “I feel left out” and “I feel isolated from others” describe how they feel, with response options based on a five-point Likert-type scale (1 = Never, 5 = Always). An aggregate variable was created by summing the two items and calculating the mean score. The new variable showed a high reliability score (α = 0.91).

#### 2.1.4. Sociodemographic Characteristics

Sociodemographic characteristics included in analyses were categorized as follows: sex (female, male); age (continuous); race/ethnicity (Hispanic; non-Hispanic Black; non-Hispanic White; Non-Hispanic Other Race); parent marital status (married; divorced, widowed, or separated; never married; member of an unmarried couple); parental education level (continuous, less than a high school degree, a high school degree or GED, some college but no college degree, 4-year college degree or higher); parent hours spent at work (continuous); and the number of kids in the household (continuous). Parents of adolescents in our sample who completed the FLASHE questionnaire and reported not working were categorized as spending zero hours at work.

### 2.2. Statistical Analyses

All statistical analyses were conducted using IBM SPSS Statistics 28. Descriptive statistics were ascertained to describe sociodemographic characteristics in our models from the FLASHE sample. We generated bivariate correlations, as well as bivariate and multivariable linear regression models, to explore the relationships between perceived parental responsiveness and their children’s: (1) emotion suppression and (2) loneliness while controlling for sociodemographic factors.

## 3. Results

### 3.1. Descriptive Statistics

Our final analytical sample size was 1576 adolescents and 1550 adolescents for our emotion suppression and loneliness models, respectively. Participants ranged from 12 to 17 years old (M = 14.48, SD = 1.61), with about half of our sample identifying as female (50.2%). A majority of participants identified as non-Hispanic White (63.7%), with 17% of our sample identifying as Non-Hispanic Black (17.0%), Hispanic (10.1%), and Non-Hispanic Other (9.2%). A total of 67.7% of adolescents in our sample had parents who are married and 39.7% of parents reported having only 1 kid in the household (See Table 1). In total, 32.9% of participants’ parents spent zero hours at work, 31.1% were at work for 31–40 h per week, 18.1% were at work for more than 41 h per week, with the remainder falling between 0 and 30 h per week at work. Based on their perceptions, most adolescents in our sample fell into the category of having responsive parents (M = 4.36, SD = 0.84) with increasing scores toward 5 indicating increased perceived parental responsiveness. Variables are reported based on the number of respondents that provided a response for each item.

As seen in Table 2, we find that perceived parental responsiveness is negatively correlated with emotion suppression, such that as perceived parental responsiveness increases, adolescents suppress their emotions less, r(1636) = −0.29, *p* < 0.001. We also find that perceived parental responsiveness is negatively associated with adolescent loneliness, such that as perceived parental responsiveness increases, adolescents are less socially isolated, r(1619) = −0.27, *p* < 0.001 (see Table 2).

### 3.2. Parental Responsiveness and Psychosocial Outcomes

A linear regression was used to examine the relationship between adolescents’ perceived parental responsiveness and their emotion suppression. An unadjusted bivariate model with adolescents’ perceived parental responsiveness as the predictor and adolescent emotional suppression as the dependent variable establishes that as parental responsiveness increases, child emotional suppression decreases (β = −0.29, *p* < 0.001). In the multivariate adjusted model (see Table 3), we find that perceived parental responsiveness significantly predicted adolescent emotion suppression (β = −0.29, *p* < 0.001), even after controlling for sex, age, race/ethnicity, parental marital status, parents’ highest education, parents’ time spent at work, and the number of kids in the household. Parents’ time spent at work and the number of kids in the household were included in addition to standard demographic variables such as marital status, age, and sex, as these may influence the amount of time parents spend with the adolescent respondent and would therefore potentially impact their perceived responsiveness. This adjusted model is able to explain 10.6% of the variance with an R-squared value of 0.106. As seen in Table 3, sex was also a significant predictor of emotion suppression (β = 0.10, *p* < 0.001), though parental responsiveness was the strongest predictor of all controlled predictor variables in the model.

A linear regression model was used to evaluate the relationship between adolescents’ perceived parental responsiveness and adolescent loneliness. An unadjusted bivariate model with perceived parental responsiveness as the predictor and adolescent loneliness as the dependent variable reveals that as parental responsiveness increases, loneliness in their children decreases (β = −0.27, *p* < 0.001). Our multivariate adjusted regression model (see Table 3) illustrates that perceived parental responsiveness was the strongest predictor for loneliness (β = −0.28, *p* < 0.001), even when controlling for sex, age, race/ethnicity, parental marital status, parents’ highest education, parents’ time spent at work, and the number of kids in the household. As indicated in Table 3, parents’ time spent at work was also a statistically significant predictor of loneliness (β = −0.11, *p* < 0.001), though parental responsiveness was the strongest predictor in the model. The adjusted model was able to explain 11.3% of the variance with an R-squared value of 0.113.

## 4. Discussion

Parenting styles are associated with various health outcomes among adolescents, such as fruit and vegetable consumption, physical activity, and screen time [38,41]. The present study explored the relationship between perceived parental responsiveness, one important facet of parenting style, and adolescent emotional suppression and loneliness. As hypothesized, perceived parental responsiveness was a significant predictor of increased adolescent emotional suppression and increased adolescent loneliness.

Study results found that adolescents who perceived they had highly responsive parents suppressed their emotions significantly less than teens who perceived they had less responsive parents. This finding is consistent with research stipulating that parents influence how their child regulates their emotions throughout their development [42]. Adolescence also canonically marks a critical time for the development or amplification of psychopathology that may arise from pre-existing maladaptive emotion regulation strategies [16,43,44,45]. Thus, it is imperative that parents are responsive to their child’s needs as a preventative and protective measure against potential negative health outcomes that arise from increased emotion suppression. This is important to note as a previous FLASHE study found that emotion suppression is associated with negative behavioral consequences in close relationships, such as emotional eating in both the parent and adolescent [46]. Furthermore, another study found emotion suppression to be positively associated with adolescent screen time and authoritative parenting, of which high responsiveness is a key characteristic. Authoritative parenting was more effective in limiting adolescent screen time when compared with other parenting styles [47]. Emotion suppression not only holds psychopathological and social implications for adolescent development but also motivates unhealthy behaviors, particularly when it comes to eating and nutrition [46,48].

The present findings suggest that the relationship between perceived parental responsiveness and emotion suppression in adolescence may be deeply entwined. The relationship observed in our results may be attributed to the fact that emotion suppression may play a role in weakening relationships, such that as an adolescent begins to suppress their emotions, it may damage their relationship with their parent, leading to more suppression and an erosion of the parent–child relationship [25,49]. Applying Ferrer et al.’s [46] model of the bidirectional nature of the parent–adolescent relationship, emotion suppression may be operating as a positive feedback loop, perpetuating this cycle where suppression leads to less social support from the parent which, in turn, fuels the mechanisms promoting further suppression moving forward. The cross-sectional nature of the present study prohibits claims of directionality; thus, future research should test this idea and explore the interactions among these relationships.

We also found that adolescents who perceived they had highly responsive parents were significantly less lonely than teens who perceived they had less responsive parents. This finding is consistent with extant research that perceived parental warmth—both paternal and maternal—is associated with lower levels of loneliness in their children [50,51]. Our findings also parallel research maintaining that greater perceptions of parental support may act as a protective factor against loneliness as scholars have found it to alleviate both the onset and the development of loneliness [52]. This study extends current literature demonstrating that greater perceived responsiveness from parents is linked to decreased loneliness. These findings can be applied to existing research which has found that parental support attenuates any adverse consequences of loneliness while also diminishing the risk of having diagnosed depression and lower self-rated health [5]. Moreover, increased perceived parental support is also recommended into emerging adulthood to help circumvent potential adverse health outcomes and enhance well-being during this transition to adulthood [48,53].

Overall, our findings suggest that greater parental responsiveness may lead to improved social and emotional well-being among adolescents. Research has shown that loneliness and emotion suppression in adolescents can lead to unintended health consequences, such as increased psychopathology and maladaptive emotional coping strategies later in life [18,28]. Thus, the present study further highlights the importance of considering the role of parents in developing health promotion interventions, particularly how parental skill education to improve connection can enhance interventions [38,54,55,56]. This aligns with existing research suggesting that interventions that target the quality of the parent–adolescent relationship and work to change unsuitable parenting styles may be particularly impactful to strengthen parents’ influence on their teen’s health behavior [57,58].

The present research is subject to limitations in both data collection and analysis. While the FLASHE dataset is large, it is cross-sectional and therefore changes over time and causality cannot be determined. Future research should consider taking a longitudinal approach, which would provide greater insight into how parental responsiveness predicts psychosocial outcomes in adolescents over time. Constructs were also only measured with a single item instead of full scales, though this is a standard limitation in national surveys. The present findings may also have some cross-cultural and generalizability limitations as the race categories available in the FLASHE data are not robust. In the coding process, FLASHE data inhibits individual analyses for Asian, Pacific Islander, and Native participants by grouping them into an ‘other race’ category. In addition to the lack of diverse representation of significant racial and ethnic groups, there is also a high percentage of White participants. Thus, future research should explore the differing roles perceived parental responsiveness may take in emotional well-being across different identities and in the context of diverse cultural backgrounds.

Despite these limitations, the study findings meaningfully contribute to the literature by demonstrating strong associations between perceived parental responsiveness and the emotional well-being of adolescents using a national sample, with greater implications for generalizability. Together, our findings suggest that implementing strategies to help parents cultivate responsive behaviors may be beneficial to improving emotional well-being in their children during adolescence and beyond. An intervention designed to support the parents of adolescents in their responses to their children’s emotions showed positive impacts on parental stress, parent–child relationship quality, and reductions in adolescent depression [59]. Further research on the effectiveness of interventions to support parents on how to respond to their adolescents is warranted.

## Figures and Tables

**Table 1:** Sociodemographic statistics for adolescents in the 2014 FLASHE survey.

Characteristic	%	Mean (SD), Range
Sex (n = 1678)		
Female	50.2	
Male	49.8	
Age (n = 1682)		14.48 (1.61), 12–17
12 years old	13.3	
13 years old	20.0	
14 years old	16.6	
15 years old	18.1	
16 years old	19.7	
17 years old	12.2	
Race/ethnicity (n = 1666)		
Non-Hispanic Other	9.2	
Hispanic	10.1	
Non-Hispanic Black	17.0	
Non-Hispanic White	63.7	
Parent Marital Status (n = 1779)		
Member of an unmarried couple	5.7	
Married	71.5	
Divorced, widowed, or separated	13.0	
Never Married	9.8	
Parent Highest Education (n = 1787)		3.27 (0.78), 1–4
Parent Time Spent at Work (n = 1839)		1.66 (2.41), 0–4
# of Kids in Household (n = 1789)		1.84 (0.78), 1–3

**Table 2:** Variable means, standard deviations, and bivariate correlation for adolescents in the 2014 FLASHE survey.

Measure	N	Mean	SD	1	2	3	4
1) Perceived Parental Responsiveness	1683	4.36	0.84	1			
2) Emotion Suppression	1657	2.75	0.93	−0.29 ***	1		
3) Loneliness	1656	2.10	1.01	−0.27 ***	0.27 ***	1	
4) Age	1682	14.48	1.61	−0.01	0.08 ***	−0.02	1
5) Parent Highest Education	1787	3.27	0.78	−0.01	−0.02	−0.02	0.02
6) Parent Time Spent at Work	1839	1.66	2.41	0.03	−0.03	−0.09 ***	0.04
7) # of Kids in Household	1789	1.85	0.78	−0.02	−0.03	−0.03	−0.10 ***

Note. *** indicates *p* is significant at 0.001.

**Table 3:** Adjusted linear regression models examining associations between parental responsiveness and psychosocial factors.

Variable	Emotion Suppression (n = 1576)					Loneliness (n = 1550)				
	b	B	SE(B)	t	*p*	b	B	SE(B)	t	*p*
Parental Responsiveness	**−0.322**	**−0.291**	**0.027**	**−12.07**	**<0.001**	**−0.333**	**−0.277**	**0.029**	**−11.44**	**<0.001**
Sex										
Female (ref)										
Male	**0.179**	**0.096**	**0.045**	**3.99**	**<0.001**	**−0.146**	**−0.072**	**0.049**	**−2.99**	**0.003**
Age	0.042	0.073	0.014	3.02	0.003	−0.020	−0.031	0.015	−1.29	0.196
Race/ethnicity										
Non-Hispanic Other (ref)										
Hispanic	−0.116	−0.037	0.102	−1.14	0.255	−0.048	−0.014	0.111	−0.43	0.665
Non-Hispanic Black	−0.112	−0.045	0.092	−1.21	0.226	−0.189	−0.069	0.101	−1.87	0.061
Non-Hispanic White	−0.131	−0.067	0.078	−1.67	0.096	0.147	0.07	0.086	1.72	0.086
Parent Marital Status										
Member of an unmarried couple (ref)										
Married	−0.067	−0.032	0.099	−0.68	0.496	0.121	0.054	0.106	1.15	0.251
Divorced, widowed, or separated	0.097	0.034	0.115	0.84	0.399	0.21	0.068	0.123	1.7	0.089
Never Married	−0.047	−0.015	0.121	−0.39	0.697	0.169	0.049	0.13	1.29	0.196
Parent Highest Education	−0.005	−0.004	0.030	−0.18	0.858	−0.003	−0.002	0.032	−0.08	0.935
Parent Time Spent at Work	−0.016	−0.03	0.013	−1.21	0.228	**−0.053**	**−0.090**	**0.015**	**−3.64**	**<0.001**
# of Kids in Household	−0.011	−0.009	0.029	−0.39	0.7	−0.059	−0.046	0.031	−1.86	0.063

## Data Availability

The data that support the findings of this study are openly available from the National Cancer Institute via the FLASHE website at cancercontrol.cancer.gov/brp/hbrb/flashe.html (accessed on 17 July 2022).
